# Infective Endocarditis Caused by *Pseudomonas stutzeri*: A Case Report and Literature Review

**DOI:** 10.3390/idr12030020

**Published:** 2020-12-02

**Authors:** Marwan J. Alwazzeh, Feras A. Alkuwaiti, Moammer Alqasim, Sarah Alwarthan, Yasser El-ghoneimy

**Affiliations:** 1 Department of Internal Medicine, King Fahd Hospital of the University, Imam Abdulrahman Bin Faisal University, King Faisal Road, Dammam 34212, Saudi Arabia; feras_a_k@yahoo.com (F.A.A.); moammeralqasim@gmail.com (M.A.); smalwarthan@iau.edu.sa (S.A.); 2 Department of General Surgery, King Fahd Hospital of the University, Imam Abdulrahman Bin Faisal University, King Faisal Road, Dammam 34212, Saudi Arabia; yfarag@iau.edu.sa

**Keywords:** *Pseudomonas stutzeri*, infective endocarditis, Saudi Arabia

## Abstract

*Pseudomonas* bacteria are widespread pathogens that account for considerable infections with significant morbidity and mortality, especially in hospitalized patients. The *Pseudomonas* genus contains a large number of species; however, the majority of infections are caused by *Pseudomonas aeruginosa*, infections by other *Pseudomonas* species are less reported. *Pseudomonas stutzeri* is a ubiquitous Gram-negative bacterium that has been reported as a causative agent of some infections, particularly in immunocompromised patients but has rarely been reported as a cause of infective endocarditis. Here, we report a case of a 55-year-old female with no significant medical history who presented with exertional dyspnea, productive cough, and fever. She was diagnosed as a case of acute anterior ST myocardial infarction, underwent double valve replacement surgery, and was found to have infective endocarditis caused by *Pseudomonas stutzeri*.

## 1. Case Report

A 55-year-old Saudi female, medically free, presented to the emergency room complaining of exertional dyspnea, New York Heart Association (NYHA) Class 2 accompanied by productive cough with whitish sputum. She had subjective fever associated with chills and rigors that was not relieved by antipyretics. She had no chest pain, palpitation, lower limb edema, orthopnea, paroxysmal nocturnal dyspnea, hemoptysis, gastrointestinal symptoms, or urinary symptoms. In addition, she denied any history of contact with sick patients, recent upper respiratory tract infection, or recent travel.

On physical examination, the patient was conscious, alert, and oriented. She was in respiratory distress; her vital signs were as follows: body temperature, 38.1 °C; pulse rate, 101 beats/minute; blood pressure, 107/52 mmHg; respiratory rate, 32 breaths/minute; and oxygen saturation, 88% breathing on room air. Cardiovascular examination revealed a pan systolic murmur over the apical area. Electrocardiography showed changes consistent with anterior ST myocardial infarction.

Laboratory investigations on admission revealed a high troponin level (83.439 ng/mL). Hematological examinations were as follows: a white blood cell count of 15,000/mcL (neutrophils 82.4%; lymphocytes 9.0%; monocytes 8.1% and eosinophils 0.1%); hemoglobin 8.2 g/L; mean corpuscular volume 87.4 fL; and platelet 98,000/mcL.

Erythrocyte sedimentation rate was 25 mm/h, C-reactive protein 13.8 mg/dL, procalcitonin 0.14 ng/mL and lactic acid 2 mmol/L. Estimation of electrolytes showed; sodium 143 mEq/L, potassium 4.30 mEq/L and bicarbonate 22 mmol/L. Liver function test showed; lactate dehydrogenase 597 U/L, otherwise unremarkable. Human immunodeficiency virus screening was negative.

Coronary angiography showed a totally occluded left anterior descending (LAD) artery at its mid to distal segment. Successful wiring was done, but no achievement of flow even with aspiration catheter with thrombolysis in myocardial infarction (TIMI) grade 1 flow. Normal very dominant right coronary artery (RCA), and normal left circumflex artery.

**Figure 1:**
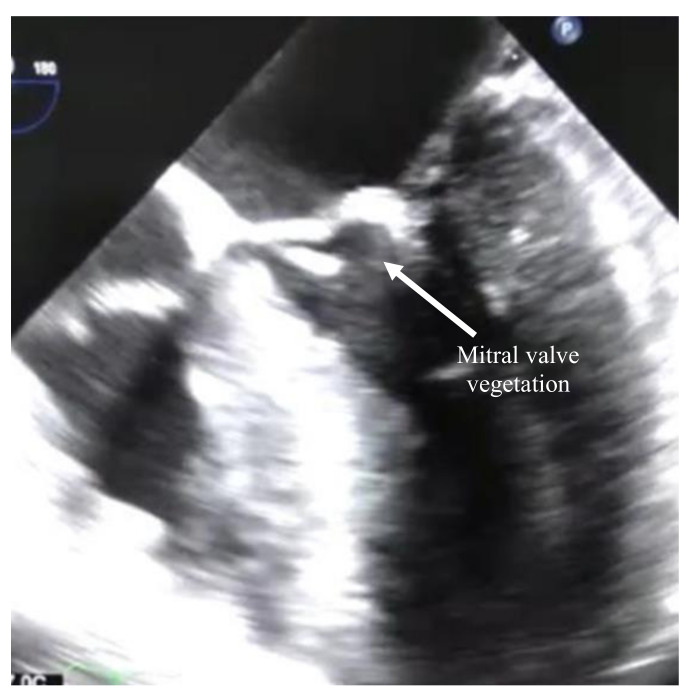
Echocardiography showing the mitral valve with a vegetation attached to the ventricular surface of the anterior leaflet (arrow).

**Figure 2:**
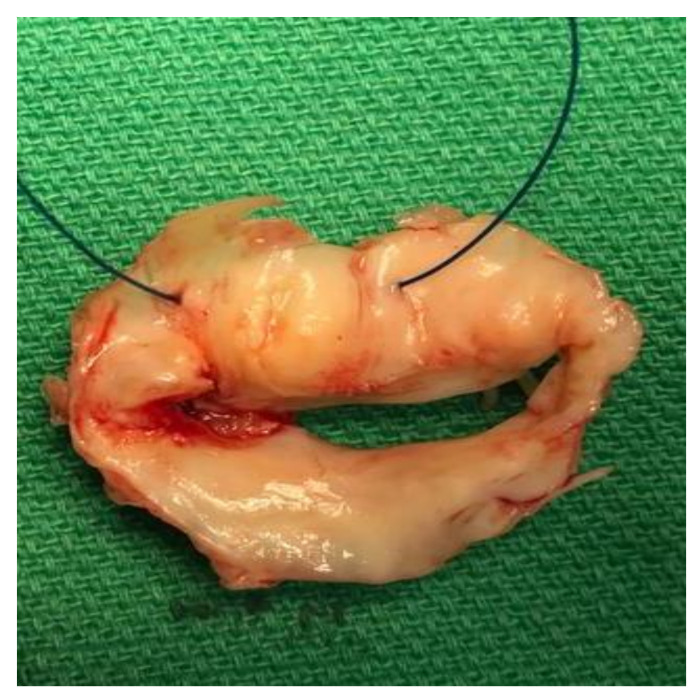
A picture of the excised mitral valve showing vegetations on the anterior leaflet and posterior commissure. No leaflet perforation.

Echocardiography was done three hours post coronary angiography and showed the mitral valve with degeneration, moderately sized vegetation attached to the ventricular surface of the anterior leaflet (6 mm × 7 mm), and severe mitral regurgitation (Figure 1). The aortic valve showed moderate leaflet thickening, mild calcified trileaflet aortic valve, large-sized vegetation attached to the left coronary cusp (16 mm × 7 mm). Therefore, the patient was diagnosed with possible infective endocarditis (IE) according to the Modified Duke Criteria (MDC) for further workup including blood cultures and empirical antibiotic treatment was started (vancomycin 1 g IV twice daily and ceftriaxone 2 g IV once daily). Then, the cardiac surgeon recommended proceeding with double valve replacement surgery. Day 5 post-admission, a double replacement was made, the left atrium was found dilated, the aortic valve severely regurgitant, and leaflets were fibrosed with calcification at the annulus. In addition, the mitral valve was severely regurgitant, the commissures destructed and the leaflet fibrosed (Figure 2). A large vegetation was also found on the anterior leaflet. Four tissue samples were sent for microbial culture. Empirical antibiotic treatment continued with minimal improvement after 8 days. The blood cultures did not show any significant growth; however, two of the tissue cultures were positive for *Pseudomonas stutzeri (P. stutzeri)*. The diagnosis of infective IE was confirmed according to the MDC (pathological). The organism was sensitive to ciprofloxacin, ceftazidime and cefepime; antibiotic treatment shifted to cefepime 2 g IV thrice daily. After five days, the patient started to improve clinically, and the inflammatory markers decreased. Six weeks of cefepime course were completed, the laboratory findings including cardiac biomarkers normalized, and ECG showed only residual ST elevations in V2 and V3. The patient was discharged home in good condition and remained well over a follow-up period of 10 months.

## 2. Discussion and Conclusions

*P. stutzeri*, which was first described by Burri and Stutzer [1], is widely distributed in the environment. When isolated, it is usually considered as colonization or contamination, but it has also been reported as a causative agent of infections with different presentations. It has been reported to cause pneumonia, meningitis, ocular infection, bacteremia, osteomyelitis and joint infections [1,2,3]. In addition, it has also been isolated as an opportunistic pathogen [2,4]. Interestingly, most cases of infection due to *P. stutzeri* have been reported from the Mediterranean Basin, suggesting that geographical factors [3]. However, this assumption should be taken with caution as there may be reporting bias or underreporting of *P. stutzeri* infections in other parts of the world.

Gram-negative bacteria are responsible for ~10% of all endocarditis cases [5]. The most frequently isolated causative pathogen in this group *is Pseudomonas aeruginosa*. *P. stutzeri* is rarely reported as a causative agent of IE, with only five such cases have been reported previously (Table 1). Of these, four patients had a prosthetic valve, in contrast to our case [3,6,7,8,9].

Myocardial infarction is a rare presentation of IE, the pathogenesis of ischemic events in IE is usually related to a non-atherosclerotic process. Historically, IE has been reported as a cause of significant coronary embolism in 1.5% of cases, and coronary artery microemboli were found to be present in more than 60% of cases on postmortem examination [10].

Positive blood culture was documented as a diagnostic criterion in the majority of IE cases caused by *P. stutzeri*. This organism shows optimal growth on peptone or yeast agar, as well as, it is capable of growing on other solid media, liquid media, and even potatoes [11]. Therefore, properly performed and processed blood culture remains an acceptable diagnostic tool for various infections caused by *P. stutzeri,* including IE. The rarity of infections caused by *P. stutzeri* can be explained by the low infectivity and pathogenicity of such indolent opportunistic pathogen, especially in immunocompetent patients.

A definitive diagnosis of IE was made in our case pathologically (positive bacterial cultures of valvular vegetations) according to MDC; however, because of its poor sensitivity and low positive predictive value, valve cultures should be interpreted with caution as a single pathological criterion of a definitive diagnosis of IE [12]. Molecular diagnosis of endocarditis by PCR can help to reduce the uncertainty and increase the sensitivity in such cases [12,13].

Four of the five previous documented cases of IE caused by *P. stutzeri* affected the prosthetic valve, while one case was documented as native valve IE. Four patients were successfully treated with antibiotics and/or surgery; however, the source of infection could not be identified. Late Prosthetic valve endocarditis was documented in Shalabi et al. and Rosenberg et al. cases [4,6]. An indolent clinical course was also well documented in the Grimaldi et al. case with relapse after four years [8]. This indicates the need for a prolonged period of follow-up of IE caused by *P. stutzeri.*

Our case is the first reported case of an IE caused by *P. stutzeri* in Saudi Arabia. The patient was immunocompetent, had no known risk factors, but the presence of fibrotic changes in mitral and aortic valves indicate an underlying pathology such as past rheumatic fever, which was prevalent in the past century in Saudi Arabia [14]. None of the previously mentioned risk factors apply to our case, including geographical relevance. The source of infection and portal of entry was unidentified in the past five cases as well as in our case.

## Figures and Tables

**Table 1:** Past case reports of infective endocarditis (IE) caused by *P. stutzeri*.

Cases	Country	Year	Valve/Type	Antibiotics and Duration	Surgery	Outcome	Time after Cardiac Surgery
Rosenberg et al. [6]	Israel	1987	Mitral/prosthetic	Tobramycin and mezlocillin for 28 days, then mezlocillin for 14 days	Not done	Cured	2 years
Lopez et al. [7]	Spain	2002	Aortic valve/native	aztreonam for 6 weeks	Aortic replacement	Cured	Not applicable
Grimaldi et al. [8]	France	2008	Aortic/Prosthetic	1st cefotaxime 1 month then ceftriaxone 1 month 2nd cefotaxime 14 days, ciprofloxacin and doxycycline for 16 months	Not done	Cured	6 and 10 years
Shalabi et al. [3]	Lebanon	2017	Aortic and mitral/prosthetic	Ceftazidime 8 weeks	Aortic replacement	Cured	3 year
Halabi et al. [9]	Lebanon	2018	Aortic/Prosthetic	Ceftazidime for a couple of days	Aortic and tricuspid valve replacement	Deceased	26 days
